# The effectiveness and cost‐effectiveness of community‐based support for adolescents receiving antiretroviral treatment: an operational research study in South Africa

**DOI:** 10.1002/jia2.25041

**Published:** 2018-02-27

**Authors:** Geoffrey Fatti, Debra Jackson, Ameena E Goga, Najma Shaikh, Brian Eley, Jean B Nachega, Ashraf Grimwood

**Affiliations:** ^1^ Kheth'Impilo Cape Town South Africa; ^2^ The South African Department of Science and Technology/National Research Foundation (DST‐NRF) Centre of Excellence in Epidemiological Modelling and Analysis (SACEMA) Stellenbosch University Stellenbosch South Africa; ^3^ UNICEF New York NY USA; ^4^ School of Public Health University of the Western Cape Cape Town South Africa; ^5^ Health Systems Research Unit South African Medical Research Council Pretoria South Africa; ^6^ Department of Paediatrics University of Pretoria Pretoria South Africa; ^7^ Department of Paediatrics and Child Health Red Cross War Memorial Children's Hospital University of Cape Town Cape Town South Africa; ^8^ Departments of Epidemiology, Infectious Diseases and Microbiology University of Pittsburgh Graduate School of Public Health Pittsburgh PA USA; ^9^ Department of Medicine and Centre for Infectious Diseases Faculty of Medicine and Health Sciences Stellenbosch University Cape Town South Africa; ^10^ Departments of Epidemiology and International Health Johns Hopkins University Bloomberg School of Public Health Baltimore MD USA

**Keywords:** HIV, antiretroviral treatment, adolescents, United Nations Sustainable Development Goals, community‐based support, cost‐effectiveness

## Abstract

**Introduction:**

Adolescents and youth receiving antiretroviral treatment (ART) in sub‐Saharan Africa have high attrition and inadequate ART outcomes, and evaluations of interventions improving ART outcomes amongst adolescents are very limited. Sustainable Development Goal (SDG) target 3c is to substantially increase the health workforce in developing countries. We measured the effectiveness and cost‐effectiveness of community‐based support (CBS) provided by lay health workers for adolescents and youth receiving ART in South Africa.

**Methods:**

A retrospective cohort study including adolescents and youth who initiated ART at 47 facilities. Previously unemployed CBS‐workers provided home‐based ART‐related education, psychosocial support, symptom screening for opportunistic infections and support to access government grants. Outcomes were compared between participants who received CBS plus standard clinic‐based care versus participants who received standard care only. Cumulative incidences of all‐cause mortality and loss to follow‐up (LTFU), adherence measured using medication possession ratios (MPRs), CD4 count slope, and virological suppression were analysed using multivariable Cox, competing‐risks regression, generalized estimating equations and mixed‐effects models over five years of ART. An expenditure approach was used to determine the incremental cost of CBS to usual care from a provider perspective. Incremental cost‐effectiveness ratios were calculated as annual cost per patient‐loss (through death or LTFU) averted.

**Results:**

Amongst 6706 participants included, 2100 (31.3%) received CBS. Participants who received CBS had reduced mortality, adjusted hazard ratio (aHR) = 0.52 (95% CI: 0.37 to 0.73; *p *< 0.0001). Cumulative LTFU was 40% lower amongst participants receiving CBS (29.9%) compared to participants without CBS (38.9%), aHR = 0.60 (95% CI: 0.51 to 0.71); *p* < 0.0001). The effectiveness of CBS in reducing attrition ranged from 42.2% after one year to 35.9% after five years. Virological suppression was similar after three years, but after five years 18.8% CBS participants versus 37.2% non‐CBS participants failed to achieve viral suppression, adjusted odds ratio = 0.24 (95% CI: 0.06 to 1.03). There were no significant differences in MPR or CD4 slope. The cost of CBS was US$49.5/patient/year. The incremental cost per patient‐loss averted was US$600 and US$776 after one and two years, respectively.

**Conclusions:**

CBS for adolescents and youth receiving ART was associated with substantially reduced patient attrition, and is a low‐cost intervention with reasonable cost‐effectiveness that can aid progress towards several health, economic and equality‐related SDG targets.

## 1 Introduction

The UN Sustainable Development Goals (SDGs) are 17 universal, ambitious and interrelated goals established to guide the development policy and agenda of member states till 2030 1. UNAIDS has also set ambitious HIV treatment targets to help end the AIDS epidemic by 2030 (SDG 3.3) 2. For the SDGs to be achievable, evidence‐based interventions need to be implemented 3, and to reach the UNAIDS treatment goals, innovative and efficient healthcare service delivery models are required 4.

Amongst adolescents in sub‐Saharan Africa (SSA), progress towards the SDGs and HIV prevention and care goals are particularly lagging 5, 6. Adolescents in SSA have the highest HIV incidence globally 7, 8, and adolescents are the only demographic group in whom AIDS‐related mortality is increasing, having tripled since 2000 9, 10. Adolescents and youth receiving antiretroviral treatment (ART) have poorer patient retention and treatment outcomes than adults 11, 12, 13, 14, 15. Ensuring high retention is a crucial aspect of the ART programme to maximize treatment outcomes 16, as well as to reduce community viral load to prevent horizontal transmission 17, 18. ART programmes retention in SSA is poor, being only 56% after five years 19. The barriers to retention amongst adolescents and youth are numerous and diverse, and include the burden of multiple vulnerabilities, barriers to healthcare access, mental health needs, a lack of psychosocial support, a lack of trained healthcare workers focusing on adolescent‐specific care, and lack of support during the transition from paediatric to adult care 20, 21, 22, 23. Appropriate, individualized, holistic and durable interventions that support adolescent's clinical, psychosocial and nutritional care have been suggested 20, 21, 23.

In SSA, adolescents and youth form the greatest proportion of the population (over 33%), and SSA is the only region in which this group continues to grow substantially 24. The health of adolescents is crucial that they may meaningfully contribute to the economy 25, 26. Their economic potential will support progress towards SDGs 1, 2, 8 and 9 to reduce poverty and hunger, promote economic growth and build industry. As SSA has very high HIV prevalence amongst adolescents and youth 27, promoting the health of adolescents and youth living with HIV is essential for the region to make meaningful progress towards the SDGs over and beyond health‐related SDGs.

HIV‐infected adolescents are a neglected group 28. Recent systematic reviews indicate that the evidence base for adherence and retention‐enhancing interventions amongst HIV‐infected adolescents and youth is very sparse, and that most studies focussed on high‐income countries and had low participant numbers 23, 28, 29. These reviews conclude that identifying effective interventions that improve ART outcomes amongst adolescents is overdue. Evidence of the longer‐term effectiveness and cost‐effectiveness of adherence and retention‐enhancing interventions are particularly lacking 30. The limited evidence that exists suggests that interventions that include individualized psychosocial support, counselling and education, and the provision of specific adolescent‐tailored services are promising and require further investigation 23, 28, 29.

SSA also has critical shortages of professional healthcare workers–particularly aggravated due to the HIV/AIDS epidemic–and needs to substantially increase its health workforce to attain its development goals 31, 32. SDG target 3c is to substantially increase the recruitment, development and training of the health workforce in developing countries 1. Community‐based support (CBS) programmes are task‐shifting healthcare interventions involving lay healthcare workers that have been developed to increase the health workforce at limited cost in developing countries 33, 34. Amongst others, CBS programmes have aimed to support HIV‐infected adults receiving ART 35. The effectiveness of CBS for adolescents receiving ART requires evaluation, and cost‐effectiveness evaluations of CBS are lacking 36.

South Africa has the greatest number of people living with HIV globally, and is showing poor performance regarding its HIV‐related SDG target 3, 37. South Africa also has one of the most unequal societies worldwide 38. South Africa's unemployment rate (27%) is amongst the ten highest national unemployment rates globally, 39, 40 with youth unemployment being approximately 50% 41. Almost two‐thirds of young South African children live in poverty, and 20% of the population live in extreme poverty 38, 42.

This study aimed to evaluate the effectiveness and cost‐effectiveness of a large CBS programme for HIV‐infected adolescents and youth receiving ART (with five years of patient outcomes) in four South African provinces.

## 2 Methods

A retrospective cohort study was performed at 47 public ART facilities, using routinely collected clinical data. The facilities were located in KwaZulu‐Natal, Western Cape, Eastern Cape and Mpumalanga provinces, in both urban (33 facilities) and rural areas (14 facilities). Included facilities were all facilities supported by Kheth'Impilo, a non‐profit organization, which had a CBS programme for adolescents and youth. Kheth'Impilo supports the South African Department of Health with public health systems strengthening. The majority were primary healthcare facilities, and six were secondary‐level hospitals. Antenatal HIV prevalence in these provinces varied between 18.2% and 37.4% 43. Co‐infection with tuberculosis amongst adolescents and youth starting ART in South Africa is high (9% to 13%) 13.

Antiretroviral‐naive adolescents and youth aged 10 to 24 years who initiated ART between 01 January 2004 and 30 September 2010 were included. Follow‐up was until mortality, loss to follow‐up (LTFU), documented transfer‐out to other sites, 30 September 2011 (database closure) or five years on ART (whichever occurred first). To evaluate the effectiveness of CBS, ART outcomes were compared between adolescents and youth who received CBS plus standard clinic‐based care versus adolescents and youth who received standard care only. During the pre‐ART preparation period, patients initiating ART were evaluated by a facility‐based community co‐ordinator (named a “site‐facilitator”), who assigned patients in a non‐randomized manner to receive CBS in addition to usual care if the following criteria were fulfilled: CBS‐workers were active in the area of the patient's home, CBS‐worker capacity was available, and patient consent was obtained. As the development of the CBS programme was progressive, few patients initially received CBS but this increased as the programme expanded. Clinical and socioeconomic factors were not criteria in the allocation of patients to receive CBS. For analyses, patients were assigned to the CBS group if they were allocated to and received support from a named CBS‐worker from ART initiation.

### 2.1 CBS intervention

CBS‐workers are clinic‐linked, lay community health workers who provided ART patient support by undertaking home visits to ascertain and address household challenges impacting on clinic attendance and adherence. CBS‐workers resided in low socioeconomic, high HIV‐prevalence areas. Preference was given to employing previously unemployed people as CBS‐workers. They were trained regarding HIV and tuberculosis (TB) infection and treatment, including addressing psychosocial issues impacting adherence. Support started from the time of pre‐ART preparation and continued throughout long‐term care. Patient, family and household issues assessed by CBS‐workers included nutrition security, substance abuse, mental health including depression, domestic violence, non‐disclosure, and HIV‐related stigma and discrimination. Issues were discussed at clinic multidisciplinary team meetings and interventions agreed by the team were implemented by the CBS‐worker as appropriate. CBS included providing one‐on‐one counselling regarding adherence, and support and referral for psychosocial problems and nutrition security. Participants were provided with information and education regarding sexual and reproductive health and family planning. Adolescents' carers were offered educational sessions regarding HIV/TB information, medication adherence, and nutrition. Adolescents and youth who defaulted clinic visits were traced by CBS‐workers. Eligibility for government social assistance grants (for poverty relief) was assessed and support provided to obtain these where eligible.

Participants were scheduled for weekly visits during the first months following ART initiation, then monthly for at least six months. Once stable, home visits were performed at least quarterly, but if clinic visits were delayed, home visit frequency increased. Health promotion education and symptom screening for TB, opportunistic infections and sexually transmitted infections (STIs) were performed, with referral to clinics for further management if indicated.

CBS‐workers had a specific geographic area which they supported and were assigned 80 to 120 patients each. Career development of CBS‐workers was encouraged, with certain CBS‐workers subsequently employed as social auxiliary workers or home‐based care co‐ordinators 44.

### 2.2 Outcomes and definitions

The primary outcomes were as follows: time to all‐cause mortality after starting ART, and time till LTFU after starting ART. Attrition was defined as a combined endpoint due to patient losses due to either mortality or LTFU. The secondary outcomes were as follows: (i) Adherence to ART measured using Medication Possession Ratios (MPRs)–an adherence measure derived from pharmacy refill data (number of days of dispensed medication divided by the number of days between the first and last pharmacy refill during the study period) 45, 46; (ii) CD4 cell count increases between months 0 and 36 after starting ART; (iii) CD4 count slope (mean change in CD4 count per month) between months 0 to 6 and 6 to 60; and (iv) the proportion of patients not achieving virological suppression after three years and during the fifth year of ART. We were primarily interested in longer‐term immunological reconstitution and virological outcomes and not the initial rapid rise in CD4 count following ART initiation 47.

Deaths were recorded as reported by professional healthcare workers or family members. Patients were defined as LTFU if they were not known to have died or to have transferred out (as documented in site databases), and had no visit to the site for six months or more prior to database closure 48, 49. Patients who returned to care after treatment interruptions were considered remaining in care. The date of last contact was assigned for the outcome of mortality or LTFU in time‐to event analyses, with one day of follow‐up added for patients who did not return after initiating ART to include them in analyses. Patients documented as transferring to other facilities were censored on the last clinic visit date. Patients who did not receive CBS who missed appointments were traced by telephone or a district tracing team would visit the home where available. All patients visited the clinic at a frequency determined by clinic professional staff (generally monthly). Virological suppression was defined as viral load <400 copies/ml. Laboratory measurements were performed by the South African National Health Laboratory Service.

Individual‐level patient data were collected prospectively for programme monitoring purposes by designated site‐based data capturers at each visit using standardized custom‐designed databases, which were regularly pooled to a data warehouse, using standardized operating procedures. Site databases were designed in Microsoft Access^®^, and were used for clinical data collection and patient and clinic management. Regular data cleaning and quality control procedures were implemented.

Participant baseline characteristics were compared with medians, interquartile ranges and percentages, and binary variables were compared with risk ratios and 95% confidence intervals. Outcomes were by intention‐to‐treat ignoring changes in exposure status after ART initiation. Cumulative incidence functions were used to calculate time till mortality or LTFU, using a competing‐risks approach. Multivariable Cox regression and Fine and Grey competing‐risks regression were used to compare mortality and LTFU between patients who received and did not receive CBS, controlling for demographic, clinical and site‐related confounding. To account for clustering of observations within sites, stratified Cox regression was conducted allowing the baseline hazard for each site to vary 50, and for the competing‐risks models site was included as a fixed effect. Incidence rate ratios of attrition were calculated stratified by site, with the combined estimate calculated using Mantel–Haenszel weights.

Mean MPR was analysed using generalized estimating equations specifying for clustering within sites and using Huber–White (robust) variance estimates. MPR was also analysed as a binary variable with mixed‐effects logistic regression including site as a random intercept, using a threshold MPR of ≥95% to indicate high adherence. CD4 count increases were analysed with linear regression, and CD4 cell slopes were analysed with multilevel mixed‐effects linear regression including site and patient as random effects to account for the longitudinal nature of the data and clustering within sites. Models were adjusted for ART duration and baseline variables were included as fixed effects. Proportions of patients not achieving viral suppression were analysed using mixed‐effects logistic regression.

To impute missing baseline covariate data, multiple imputations by chained equations were conducted using 20 imputed datasets, under the assumption that missing data were likely missing at random. Multivariable analyses were run on each data set that included the imputed values and the results combined, using Rubin rules 51.

All available plausible demographic, clinical and site‐related variables were considered as potential confounders and were included in multivariable models when their inclusion altered the association between CBS and the outcomes or were significantly associated with the outcomes with *p* < 0.05. Modification of the effect of CBS on outcomes was assessed by stratifying effect measures by plausible modifiers. The number needed to treat (NNT) to prevent a case of death or LTFU were calculated as appropriate for time‐to‐event outcomes 52.

### 2.3 Cost‐effectiveness analyses

A top‐down expenditure approach was used to determine the incremental cost of CBS to usual ART care from a provider perspective. Expenditure of the CBS programme according to the financial records of the programme were collected, which included costs of human resources, training, management and administration, infrastructure and equipment, and monitoring and evaluation over a two‐year period between 01 April 2011 and 31 March 2013. The cost of usual ART patient care was not considered and was assumed to be equal between patients with and without CBS. The number of patient‐years of CBS during this period was calculated from programme monitoring data.

The cost outcomes were: (i) average cost of CBS per patient‐year of support, and (ii) cost‐effectiveness defined as cost per patient‐loss (through death or LTFU) averted. The effectiveness of CBS in preventing patient attrition at annual intervals after starting ART (compared to usual care) was calculated as the difference in patient attrition between patients who did and who did not receive CBS (estimated from a stratified Cox model) divided by attrition amongst patients who did not receive CBS 53. Incremental cost‐effectiveness ratios (ICERs) were calculated from one through five years of treatment. For cost calculations, patients lost to care were considered lost at the mid‐point of each year. Costs were converted to United States dollars at the average exchange rate of ZAR 1 = US$0.1219 in 2012 54. For ICERs, costs and patient losses averted were discounted at 3% per annum 55. Analyses were conducted with Stata^®^ version 13.1 (College Station, TX, USA), and Microsoft Excel^®^. The University of Cape Town Human Research Ethics Committee provided the studies ethical approval, and the study conformed to the Declaration of Helsinki ethical principles.

## 3 Results

Database records of 85,997 patients who initiated ART were screened for inclusion, with the following excluded: 3756 patients aged <10 years when starting ART; 74,123 aged ≥25 years; and 1412 who started ART after the study enrolment period. Thus 6706 participants were included, of whom 2100 (31.3%) received CBS and 4606 (68.7%) who received standard care only. Most (82.4%) participants were female and 1810 (27.0%) were aged 10 to 19 years. At ART initiation, participants who received CBS had: a higher proportion with advanced clinical stage disease (World Health Organization (WHO) stages III/IV), a slightly higher median CD4 count, a higher proportion who received concomitant TB treatment, a higher proportion who were pregnant, a higher proportion who attended rural facilities and a higher proportion who attended primary healthcare clinics (Table 1). The proportion of patients who received CBS increased from 19.3% to 33.5% during the study period.

**Table 1:** Characteristics of adolescents and youth at antiretroviral treatment initiation who received and did not receive CBS in South Africa

	Total (n = 6706)	Did not received CBS (n = 4606)	Received CBS (n = 2100)	Risk ratio (CBS vs. no CBS) (95% CI)^a^
Female, n (%) (n = 6706)	5523 (82.4)	3752 (81.5)	1771 (84.3)	1.04 (1.01 to 1.06)
Median age, years, (IQR) (n = 6706)	22.4 (19.6 to 23.9)	22.4 (19.5 to 23.9)	22.5 (19.9 to 23.9)	
Age categories, n (%) (n = 6706)				
Ages 10 to 19 years	1810 (27.0)	1268 (27.5)	542 (25.8)	0.93 (0.86 to 1.02)
Ages 20 to 24 years	4896 (73.0)	3338 (72.5)	1558 (74.2)	
WHO clinical stage, n (%) (n = 4424)				
I/II	1564 (35.4)	1171 (37.5)	393 (30.1)	
III/IV	2860 (64.7)	1949 (62.5)	911 (69.9)	1.12 (1.06 to 1.17)
CD4 cell count, cells/μl, median (IQR) (n = 5560)	136 (70 to 187)	131 (65 to 182)	145 (82 to 195)	
Pregnancy amongst females, n (%) (n = 5166)				
Not pregnant	4512 (87.3)	3031 (88.4)	1481 (85.3)	
Pregnant	654 (12.7)	399 (11.6)	255 (14.7)	1.26 (1.09 to 1.46)
Received tuberculosis treatment, n (%) (n = 6332)				
No	5623 (88.8)	3831 (89.6)	1792 (87.1)	
Yes	709 (11.2)	443 (10.4)	266 (12.9)	1.25 (1.08 to 1.44)
Initial regimen, n (%) (n = 5657)				
d4T‐3TC‐EFV	2792 (49.4)	1961 (52.5)	831 (43.2)	
d4T‐3TC‐NVP	2006 (1342)	1342 (36.0)	664 (34.5)	
ZDV‐3TC‐EFV	38 (0.7)	19 (0.5)	19 (1.0)	
ZDV‐3TC‐NVP	106 (1.9)	37 (1.0)	69 (3.6)	
TDF‐3TC‐EFV	339 (6.0)	163 (4.4)	176 (9.2)	
TDF‐3TC‐NVP	322 (5.7)	184 (4.9)	138 (7.2)	
Other	54 (1.0)	27 (0.7)	27 (1.4)	
Year of starting ART, n (%)(n = 6706)				
2004 to 2005	218 (3.3)	176 (3.8)	42 (2.0)	
2006 to 2007	1384 (20.6)	1038 (22.5)	346 (16.5)	
2008 to 2010	5104 (76.1)	3392 (73.6)	1712 (81.5)	
Location of site attended, n (%) (n = 6706)				
Urban	5238 (78.1)	3784 (82.2)	1454 (69.2)	
Rural	1468 (21.9)	822 (17.9)	646 (30.8)	1.72 (1.58 to 1.88)
Hospital‐based clinic/primary healthcare clinic attended, n (%) (n = 6706)				
Hospital	1612 (24.0)	1407 (30.6)	205 (9.8)	
Primary healthcare clinic	5094 (76.0)	3199 (69.5)	1895 (90.2)	1.30 (1.27 to 1.33)
Province, n (%) (n = 6706)				
Western Cape	803 (12.0)	523 (11.4)	280 (13.3)	
Eastern Cape	1259 (18.8)	587 (12.7)	672 (32.0)	
KwaZulu‐Natal	4035 (60.2)	3243 (70.4)	792 (37.7)	
Mpumalanga	609 (9.1)	253 (5.5)	356 (17.0)	

ART, antiretroviral treatment; CBS; community‐based support; WHO, World Health Organization; IQR, interquartile range; CI, confidence interval; d4T, stavudine; 3TC, lamivudine; EFV, efavirenz; NVP, nevirapine; ZDV, zidovudine; TDF, tenofovir.

a For binary variables.

**Figure 1:**
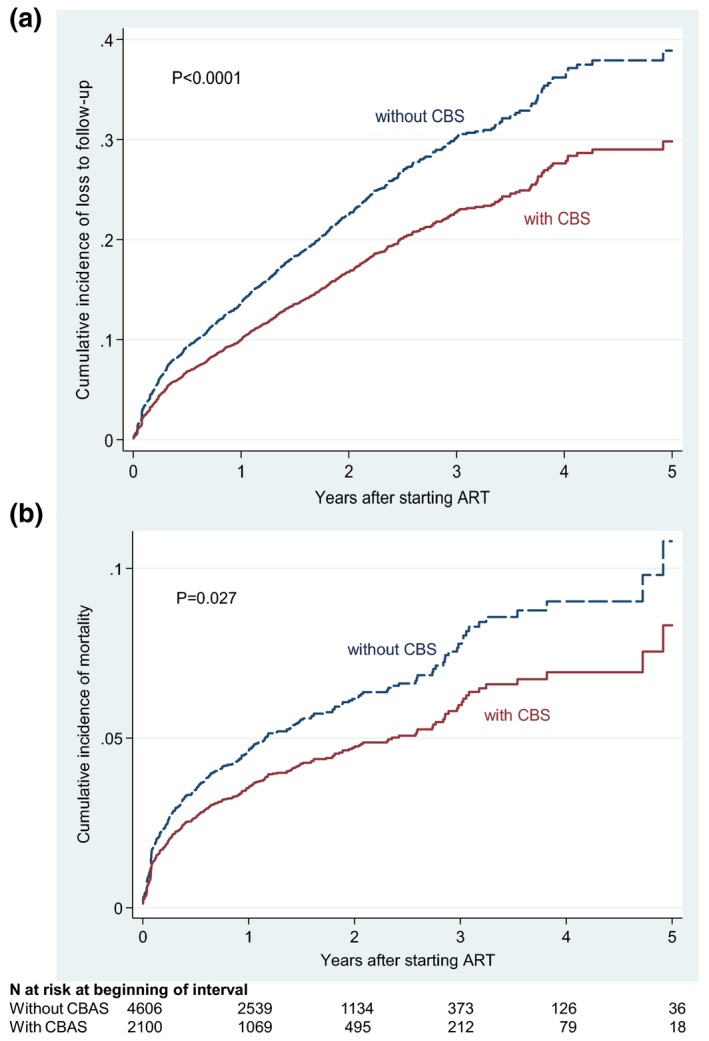
Cumulative incidence of (**A**) Loss to follow‐up and (**B**) mortality amongst adolescents and youth starting antiretroviral treatment in South Africa.

During 9215 person‐years of follow‐up, 87 (4.1%) and 256 (5.6%) of participants who received and did not receive CBS were reported as having died, respectively (*p* = 0.015). A further 286 (13.6%) and 885 (19.2%) became LTFU amongst those who received and did not receive CBS, respectively (*p* < 0.0001). 375 (8.5%) participants transferred out. After five years of ART, the cumulative incidence of mortality amongst adolescents and youth who received and did not receive CBS was 8.3% and 10.8%, respectively (*p* = 0.027), and the cumulative incidence of LTFU was 29.9% and 38.9%, respectively (*p* < 0.0001) (Figure 1).

For multivariable analyses, the proportions of imputed baseline values were: TB treatment status‐5.6%; pregnancy status‐5.3%; CD4 count‐17.1%; initial regimen‐15.6%; WHO stage‐34.0%. After controlling for confounding using multivariable Cox regression, participants who received CBS had a significantly reduced probability of mortality, adjusted hazard ratio (aHR) = 0.52 (95% CI: 0.37 to 0.73; *p* < 0.0001) (Table 2). Estimates from the competing‐risks regression models were similar. Adolescents and youth who received CBS had a 40% reduced probability of becoming LTFU, aHR = 0.60 (95% CI: 0.51 to 0.71; *p* < 0.0001). The effect of CBS on LTFU was more pronounced at rural facilities, aHR = 0.43 (95% CI: 0.30 to 0.62) and slightly more pronounced amongst pregnant women, aHR = 0.53 (95% CI: 0.31 to 0.92).

**Table 2:** Univariable and multivariable models of factors associated with loss to follow‐up and mortality amongst adolescents initiating ART in South Africa

Predictor (baseline)	Loss to follow‐up						Mortality					
	Univariable Cox		Multivariable Cox		Multivariable competing risks		Univariable Cox		Multivariable Cox		Multivariable competing risks	
	HR (95% CI)	*p*‐value	aHR (95% CI)	*p*‐value	asHR (95% CI)	*p*‐value	HR (95% CI)	*p*‐value	aHR (95% CI)	*p*‐value	asHR (95% CI)	*p*‐value
Received CBS												
Yes	0.59 (0.50 to 0.70)	<0.0001	0.60 (0.51 to 0.71)	<0.0001	0.61 (0.52 to 0.73)	<0.0001	0.45 (0.32 to 0.63)	<0.0001	0.52 (0.37 to 0.73)	<0.0001	0.56 (0.41 to 0.76)	<0.0001
No	Reference	‐	Reference	‐	Reference	‐	Reference	‐	Reference	‐	Reference	‐
Age (years)	1.03 (1.02 to 1.05)	<0.0001	1.03 (1.02 to 1.05)	<0.0001	1.04 (1.02 to 1.05)	<0.0001	1.00 (0.98 to 1.03)	0.88	0.99 (0.96 to 1.01)	0.29	0.98 (0.96 to 1.01)	0.28
Gender												
Female	Reference	‐	Reference	‐	Reference	‐	Reference	‐	Reference	‐	Reference	‐
Male	0.86 (0.73 to 1.00)	0.048	0.97 (0.82 to 1.15)	0.71	0.97 (0.82 to 1.15)	0.70	1.02 (0.78 to 1.35)	0.84	0.91 (0.67 to 1.22)	0.52	0.90 (0.33 to 1.21)	0.48
WHO stage												
I/II	Reference	‐	Reference	‐	Reference	‐	Reference	‐	Reference	‐	Reference	‐
III	1.10 (0.94 to 1.29)	0.22	1.18 (1.00 to 1.39)	0.049	1.18 (1.02 to 1.37)	0.028	2.19 (1.54 to 3.11)	<0.0001	1.84 (1.29 to 2.64)	0.001	1.86 (1.30 to 2.66)	0.001
IV	1.12 (0.86 to 1.48)	0.38	1.20 (0.91 to 1.60)	0.19	1.21 (0.93 to 1.57)	0.16	4.5 (2.79 to 7.27)	<0.0001	3.48 (2.15 to 5.62)	<0.0001	3.4 (2.12 to 5.51)	<0.0001
CD4 count, cells/μl												
0 to 99	Reference	‐	Reference	‐	Reference	‐	Reference	‐	Reference	‐	Reference	‐
100 to 199	1.06 (0.92 to 1.22)	0.40	1.04 (0.90 to 1.20)	0.63	1.09 (0.94 to 1.27)	0.25	0.36 (0.28 to 0.47)	<0.0001	0.42 (0.31 to 0.54)	<0.0001	0.42 (0.32 to 0.55)	<0.0001
200 to 349	1.11 (0.91 to 1.36)	0.30	1.11 (0.90 to 1.37)	0.32	1.17 (0.94 to 1.45)	0.17	0.27 (0.17 to 0.42)	<0.0001	0.36 (0.22 to 0.58)	<0.0001	0.35 (0.21 to 0.56)	<0.0001
≥350	1.03 (0.86 to 1.23)	0.72	1.33 (0.93 to 1.92)	0.12	1.47 (1.02 to 2.10)	0.036	0.16 (0.05 to 0.51)	0.002	0.18 (0.05 to 0.57)	0.004	0.18 (0.05 to 0.60)	0.005
Pregnancy												
Yes	1.42 (1.17 to 1.72)	<0.0001	1.43 (1.17 to 1.74)	<0.0001	1.45 (1.19 to 1.77)	<0.0001	0.25 (0.12 to 0.52)	<0.0001	0.38 (0.19 to 0.79)	0.010	0.38 (0.19 to 0.79)	0.009
No	Reference	‐	Reference	‐			Reference	‐	Reference	‐	Reference	‐
TB treatment												
Yes	0.95 (0.79 to 1.15)	0.61	0.98 (0.80 to 1.19)	0.82	0.98 (0.81 to 1.19)	0.87	1.10 (0.77 to 1.55)	0.61	0.88 (0.61 to 1.29)	0.48	0.90 (0.63 to 1.30)	0.57
No	Reference	‐	Reference	‐	Reference	‐	Reference	‐	Reference	‐	Reference	‐
Year of starting ART (continuous)	1.12 (1.05 to 1.19)	<0.0001	1.17 (1.10 to 1.25)	<0.0001	1.15 (1.08 to 1.22)	<0.0001	0.71 (0.64 to 0.79)	<0.0001	0.77 (0.69 to 0.86)	<0.0001	0.74 (0.67 to 0.82)	<0.0001
Site location												
Urban	Reference	‐	Reference	‐	Reference	‐	Reference	‐	Reference	‐	Reference	‐
Rural	1.01 (0.27 to 3.75)	0.98	1.15 (0.31 to 4.27)	0.83	0.65 (0.17 to 2.50)	0.54	1.46 (0.16 to 12.60)	0.73	1.19 (0.13 to 11.03)	0.88	1.31 (0.15 to 11.7)	0.81
PHC clinic /hospital												
Hospital	0.68 (0.51 to 0.90)	0.007	0.71 (0.53 to 0.96)	0.025	0.57 (0.25 to 1.30)	0.19	1.35 (0.77 to 2.37)	0.30	0.88 (0.47 to 1.64)	0.69	3.42 (0.40 to 28.9)	0.26
PHC clinic	Reference	‐	Reference	‐	Reference	‐	Reference	‐	Reference	‐	Reference	‐

Regression results using models with multiple imputation of missing covariate data, using 20 imputed datasets. To account for clustering within sites, Cox models were stratified by site, and a fixed‐effects approach was used for the competing risks models. Multivariable models were also adjusted for initial antiretroviral regimen. HR, hazard ratio; aHR, adjusted hazard ratio; asHR, adjusted subhazard ratio; CBS, community‐based support; ART, antiretroviral treatment; TB, tuberculosis; WHO, World Health Organization; PHC, primary healthcare; CI, confidence interval.

The NNT to prevent one case of mortality after one and three years was 6.4 (95% CI: 3.6 to 16.7) and 5.3 (3.2 to 13.0), respectively, and the NNT to prevent one case of LTFU after one and three years was 6.0 (95% CI: 4.4 to 9.4) and 5.4 (4.2 to 8.0), respectively.

Considering the combined endpoint of attrition, the incidence rate of attrition was 12.9 cases/100 person‐years (95% CI: 11.7 to 14.3) amongst adolescents and youth who received CBS, and 18.0 cases/100 person‐years (95% CI: 17.0 to 19.1) amongst adolescents and youth without CBS, incidence rate ratio (stratified by site) = 0.55 (95% CI: 0.48 to 0.64; *p* < 0.0001).

Mean MPR was similar between patients with and without CBS; 82.5% and 83.0%, respectively, adjusted mean difference = −1.0 % (95% CI: −2.6% to 0.5%), *p* = 0.20 (Table 3). There was no difference in the proportion of patients who achieved high adherence (MPR ≥95%), viz. 35.4% and 35.8% amongst patients with and without CBS, respectively, adjusted odds ratio (aOR) = 1.00 (95% CI: 0.86 to 1.19; *p* = 0.92).

**Table 3:** Secondary outcomes of CBS for adolescents and youth receiving antiretroviral treatment in South Africa

Outcome	Received CBS	Did not receive CBS	Crude effect measure (95% CI) (CBS vs. no CBS)	Crude *p*‐value	Adjusted effect measure (95% CI)^a^	Adjusted *p*‐value
Mean MPR, % (95% CI)	82.5% (81.6% to 83.4%)	83.0% (82.3% to 83.7%)	−0.6% (−1.7% to 0.6%)^b^	0.33	−1.0% (−2.6% to 0.5%)^c^	0.20
Proportion with MPR ≥95%, % (95% CI)	35.4% (33.2% to 37.6%)	35.8% (34.1% to 37.5%)	0.99 (0.92 to 1.07)^d^	0.79	1.00 (0.86 to 1.19)^e^	0.92
CD4 count increases after three years of ART, cells/μl (IQR)	384.5 (152 to 521)	366 (208 to 485)	11.9 (−67.6 to 91.6)^f^	0.76	21.8 (−60.2 to 103.9)^f^	0.60
CD4 cell slope between months 0 and 6 after ART initiation, cells/μl/month, median (IQR)	27.0 (12.9 to 43.4)	25.6 (11.9 to 42.0)	1.31 (−1.92 to 4.55)^g^	0.43	2.10 (−1.21 to 5.39)^g^	0.22
CD4 cell slope between months 6 and 60 after ART initiation, cells/μl/month, median (IQR)	6.7 (−2.0 to 16.4)	7.1 (−0.6 to 16.1)	1.09 (−1.34 to 3.51)^g^	0.38	1.28 (−1.12 to 3.68)^g^	0.30
Proportions not achieving viral suppression after three years of ART, % (95% CI)	28.2% (19.7% to 37.9%)	32.7% (26.1% to 39.7%)	0.81 (0.48 to 1.36)^e^	0.43	0.96 (0.41 to 2.28)^e^	0.93
Proportions not achieving viral suppression during fifth year of ART, % (95% CI)	18.8% (7.2% to 36.4%)	37.2% (24.1% to 51.9%)	0.39 (0.14 to 1.11)^e^	0.079	0.24 (0.06 to 1.03)^e^	0.055

a Adjusted for baseline confounding using 20 multiple imputed datasets.

b Mean absolute difference.

c Coefficient from generalized estimating equation specifying for clustering within sites.

d Risk ratio.

e Odds ratios using mixed‐effects logistic regression including site as a random intercept.

f Coefficient from linear regression.

g Coefficient from mixed‐effects linear regression (cells/μl/month) including site and individual as random effects, and adjusted for duration of ART.

CBS, community‐based support; MPR, medication possession ratios; IQR, interquartile range.

CD4 count increases were 384.5 cells/μl and 366 cells/μl amongst adolescents and youth with and without CBS, respectively, after 36 months. CD4 count slope between months 6 to 60 in adolescents and youth with and without CBS was 6.7 cells/μl/month and 7.1 cells/μl/month, respectively, with no difference in multivariable analyses; coefficient = 1.28 cells/μl/month (95% CI: −1.12 to 3.68; *p* = 0.30).

The proportions of adolescents with and without CBS who failed to achieve virological suppression after three years were similar, aOR = 0.96 (95% CI: 0.41 to 2.28), *p* = 0.93. During the fifth year of ART, the proportions with and without CBS who failed to achieve virological suppression were 18.8% and 37.2%, respectively, with the adjusted effect measure approaching a significant difference in favour of CBS, aOR = 0.24 (95% CI: 0.06 to 1.03), *p* = 0.055.

### 3.1 Cost‐effectiveness results

The average cost of CBS was US$49.5/patient/year, with 84% spent on human resources (Table 4). The entire programme employed 576 CBS‐workers. The effectiveness of CBS in reducing patient attrition ranged from 42.2% after one year to 35.9% after five years. The incremental cost of CBS per patient‐loss averted after one, two and five years was US$600, US$776 and US$1149, respectively (Table 5).

**Table 4:** Costs of CBS for antiretroviral treatment patients in South Africa

Total patient‐years supported	126,485
No. community workers employed	576
**Item**	**Average costs per patient year supported, US$ (%)** a
Human resources	41.83 (84.4)
Training	5.97 (12.1)
Infrastructure and equipment	0.02 (0.05)
Clothing for CBS‐workers	0.15 (0.3)
Management and administration	0.48 (1.0)
Monitoring and evaluation	0.10 (0.2)
Overhead costs	0.99 (2.0)
Total cost per patient supported/year	49.5 (100.0)

a Values in parentheses are percentages of the total cost.

**Table 5:** Cost‐effectiveness of CBS for ART patients in South Africa

Duration of ART (years)	Proportion of patients retained in care (%)^a^		Effectiveness of intervention in reducing patient attrition (%)^b^	No. patient losses averted due to CBS (per 100 patients initiating ART)^c^	Cumulative cost of CBS (per 100 patients initiating ART), US$^c^^,^ d	Cost‐effectiveness ratio (US$/patient‐loss averted)
	With CBS	Without CBS				
1	89.3	81.5	42.2	7.6	4549	600.7
2	82.7	71.0	40.3	11.0	8561	776.3
3	76.4	61.5	38.7	13.6	12,165	892.1
4	70.7	53.5	37	15.3	15,400	1007.7
5	66.9	48.4	35.9	16.0	18,337	1149.1

a Estimated from the survivor function of a stratified Cox model.

b The effectiveness of the CBS programme in preventing attrition (through death or loss to follow‐up) was calculated as the difference in patient attrition between patients who did and who did not receive CBS divided by attrition amongst patients who did not receive CBS.

c Costs and no. of patient losses averted were discounted at 3% per annum.

d Patients lost to the programme were considered lost at the mid‐point of each year.

CBS, community‐based support; ART, antiretroviral treatment.

## 4 Discussion

The SDGs are opportune to improve the health and wellbeing of disadvantaged groups globally. Government commitment to the SDGs needs to be translated into programmes that can deliver on the wide‐ranging goals and accompanying targets. The SDG targets are interrelated and overlap; notably 28 targets across 11 goals are health‐related 3, 26. To reach the SDGs for adolescents by 2030, the importance of innovations in adolescent health involving biomedical and behavioural interventions delivered together has recently been highlighted 56.

Adolescents are a key group for targeting of the UNAIDS 90‐90‐90 HIV treatment goals 57. In view of their poorer ART outcomes, there have previously been calls for adolescents and youth to receive specific additional support 11, 12, 13, 15. This study has found that CBS was associated with substantially improved retention in adolescents and youth receiving ART, and is a low‐cost intervention with reasonable cost‐effectiveness. Cost‐effectiveness of CBS was greatest during the first two years of treatment.

Improved programme retention increases the number of HIV‐infected adolescents and youth receiving ART, which would lead to greater numbers potentially being able to achieve viral suppression due to ART use. In turn, this can potentially decrease sexual transmission due to ART 58, 59 and aid progress towards SDG target 3.3 to reduce HIV incidence.

Community support has previously been found to reduce ART programme attrition amongst adults and children 35, 60. Mechanisms underlying these improvements include defaulter tracing, psychosocial support offered by CBS workers, improved patient links with clinics, decreased treatment fatigue, improved self‐management skills regarding HIV/AIDS, greater disclosure, greater social capital and a widened community safety net 35, 61, 62. The primary driver of decreased attrition associated with CBS in this study was reduced LTFU, with reduced mortality accounting for a small component only. Except for a trend towards improved viral suppression at five years amongst those who received CBS, significant differences in immunological restitution or the adherence measure utilized were not observed. In the absence of these, the reasons for the difference in mortality observed are unclear and require further research. It is plausible that CBS was associated with health aspects not measured in this study, such as earlier referral and treatment for incident opportunistic infections, improvements in nutritional status or mental health, or improved socioeconomic status through access to grants. Future research should also incorporate more accurate measures of adherence.

In adults, the cost‐effectiveness of strategies to reduce ART patient attrition have been evaluated in two previous studies. A hypothetical study found that interventions costing up to US$120/person/annum with effectiveness ≥40% in reducing attrition would be cost‐effective with high degrees of regional ART coverage 63. A Cote d'Ivoire study found that interventions preventing LTFU would result in a substantial saving of life‐years, and an intervention costing US$53 per person/annum would be cost‐effective by international criteria (<3 times gross domestic product per capita) if ≥28% effective 53. Although we did not model cost‐effectiveness based on disability‐adjusted life years averted, CBS was found to cost US$50/person/annum and have effectiveness between 42% to 36%, and would thus be expected to cost‐effectively reduce high attrition amongst SSA adolescents and youth.

The health workforce underpins every aspect of the health system, and is the rate‐limiting step in achieving universal health coverage by 2030 64. There is pronounced inequity in the distribution of health workers globally, with Africa carrying 25% of the world's disease burden but only 1.3% of the world's health workers, with little progress being evident in this regard 65, 66. To achieve health‐related SDGs, task‐shifting to maximize the use of available funds and health workers in the region will be essential. Efficiency and value for money will be important priorities. Amongst children, UNICEF is promoting task‐shifting from professional to community health workers to improve access to health interventions, in order to achieve SDG target 3.2 to prevent common causes of child mortality 67. The CBS programme evaluated in this study extends this model for the care of HIV‐infected adolescents and youth.

Community health workers can play a key role in attaining a number of SDGs, including health, ending poverty and hunger, equality, clean water and sanitation, and partnerships for global health (SDG 17), as highlighted in the recent Kampala statement 68, 69. Important actions to support the role of community health workers in this regard include financial and political support, partnerships with a range of healthcare providers, and disseminating cross‐country learnings. Rigorous research to expand the evidence base for policy and practice to maximize the contribution and potential of community health workers in progress towards these SDGs is vital 70. Research priorities include the roles of cross‐cutting enabling factors such as education and accreditation of community health workers, management, effective linkage with other professional staff cadres, remuneration, and motivation and performance 64, 68. Translating evidence to investment decisions will also be required to enable sustainable health solutions in pursuit of the SDGs. Including community engagement as an additional aspect of the SDG health targets has also been suggested 26.

Innovations in health worker training will be important in attaining the SDGs. CBS involves training previously unemployed persons living in impoverished areas and employing them as lay health workers, and assisting their further career development 44. As CBS is labour‐intensive, large CBS programmes will aid progress towards SDG targets 4.4, 8.5 and 8.6 (provision of skills to facilitate employment and job creation). Job‐creation further impacts other health‐related targets, as access to gainful employment improves the mental and physical well‐being of families and young people 26. Provision of jobs for CBS‐workers also increases income to the lowest 40% income group (SDG target 10.1) which can support the targets to reduce poverty and food insecurity amongst CBS‐workers and their families (SDG targets 1.1, 1.2 and 2.1).

HIV‐related interventions that have cross‐sectoral benefits produce development synergies and will accelerate progress across development goals 71. CBS‐workers provided counselling regarding mental health, sexual and reproductive health (particularly for adolescent girls), nutrition counselling, and support to access social grants. These interventions can aid progress towards SDG target 3.4 (promotion of mental health and wellbeing), SDG target 3.7 (universal access to sexual and reproductive healthcare services), as well as reduce poverty and hunger. As almost 85% of CBS‐supported participants were female, gender‐equality progress (SDG target 5.6) is also supported. The impact of these services was not assessed in this evaluation; however, future economic analyses may incorporate the potential cross‐sectoral benefits of CBS.

South Africa has recently introduced and is scaling‐up implementation of new national adherence guidelines 72. In line with this, CBS workers currently provide home and clinic‐based support for the initial 12 months after starting ART and for patients who are unstable. This study's results provide evidence of the effectiveness of an individualized approach to support adolescents and youth, and encourage scale‐up of implementation of these guidelines. Individual and group counselling and education for adolescents have shown promise in previous smaller studies conducted mostly in developed countries 28, 29. The role of CBS workers is currently expanding to include facilitation of community and clinic‐based adherence clubs for stable, virologically suppressed adults from 12 months of ART and beyond.

Challenges faced by the CBS programme include the rural context of many patients' homes with long travel distances and inadequate transport, and inconsistent availability of some adolescents for follow‐up counselling sessions. CBS is not a panacea, and other important facets of comprehensive care include youth‐friendly clinical management, peer‐support groups, and integrated management of the transition from child to adult care services 20, 21.

The strengths of this study include the large sample size drawn from many sites situated in low‐income, high HIV prevalence areas, with results thus likely being generalizable to other SSA areas. Prospectively collected individual‐level data were collected with up to five years of patient follow‐up. Additionally, clinical as well as cost outcomes were analysed.

The study limitations include the non‐random allocation of patients to groups, with the potential for selection bias and unmeasured or residual confounding. Effect measures were, however, adjusted for site‐related and individual‐level confounding using multiple imputation of missing covariate values. Differences in measured baseline characteristics were observed between CBS and non‐CBS patients; however, most confounders associated with increased attrition were more prevalent amongst CBS patients (advanced clinical stage disease 73, concurrent TB 74, pregnancy 75, more recent year of starting ART 14, 76, and attending rural facilities 77). Residual confounding is thus unlikely to have confounded effect measures in favour of CBS. The routine nature of the data may have produced information bias. Mortality was likely underestimated in both CBS and non‐CBS patients, as misclassification of patients who have died as being LTFU is common in SSA routine ART data 78. Patients who were classified as LTFU may have been undocumented transfers to other treatment sites outside the study facilities.

## 5 Conclusions

The SDG process reinforces the central importance of health in sustainable development. Greater attention to adolescent health, particular regarding HIV/AIDS, will be critical to achieve universal and sustainable development 56. This study found CBS to be a low‐cost intervention associated with substantially improved retention in adolescents and youth receiving ART, which had reasonable cost‐effectiveness. CBS for adolescents and youth can potentially aid progress towards twelve targets from eight health, economic, equality and education‐related SDGs. Future qualitative research may shed greater light on mechanisms that may improve outcomes and how community‐support may be further tailored specifically for adolescents.

## Competing interests

The authors all declare that they have no conflicts of interests.
